# In vitro evaluation of *Panax notoginseng* Rg1 released from collagen/chitosan-gelatin microsphere scaffolds for angiogenesis

**DOI:** 10.1186/1475-925X-12-134

**Published:** 2013-12-31

**Authors:** Yurong Zheng, Zhanzeng Feng, Chuangang You, Yunyun Jin, Xinlei Hu, Xingang Wang, Chunmao Han

**Affiliations:** 1 Department of Burns, Second Affiliated Hospital of Zhejiang University, School of Medicine, Hangzhou 310009, China; 2 Department of Orthopedics, Second Affiliated Hospital of Zhejiang University, College of Medicine, Binjiang Branch, Hangzhou 310000, China

**Keywords:** Angiogenesis, *Panax notoginseng* saponins, Gelatin microsphere, Controlled release, Vascular endothelial growth factor (VEGF)

## Abstract

**Background:**

The emergence of skin substitutes provides a new approach for the treatment of wound repair and healing. The consistent and steady release of angiogenic factors is an important factor in the promotion of angiogenesis in skin substitutes, which usually lack, yet need, a vascular network.

**Methods:**

In this study, ginsenoside Rg1, a natural compound isolated from *Panax notoginseng* (PNS), was incorporated into a collagen/chitosan-gelatin microsphere (CC-GMS) scaffold. The cumulative release kinetics were evaluated, and the effects of the released Rg1 on human umbilical vein endothelial cells (HUVECs) behavior, including proliferation, migration, tube formation, cell-cycle progression, cell apoptosis, and vascular endothelial growth factor (VEGF) secretion, were investigated. Additionally, HUVECs were cultured on the CC-GMS scaffold to test its biocompatibility. Standard Rg1 and VEGF were used as positive controls.

**Results:**

The results indicated that the CC-GMS scaffold had good release kinetics. The Rg1 released from the CC-GMS scaffold did not lose its activity and had a significant effect on HUVEC proliferation. Both Rg1 and VEGF promoted HUVEC migration and tube formation. Rg1 did not induce HUVEC apoptosis but instead promoted HUVEC progression into the S and G2/M phases of the cell cycle. Rg1 significantly increased VEGF secretion compared with that in the control group. HUVEC culture on the CC-GMS scaffold indicated that this scaffold has good biocompatibility and that CC-GMS scaffolds containing different concentrations of Rg1 promote HUVEC attachment in a dose- and time-dependent manner.

**Conclusions:**

Rg1 may represent a new class of angiogenic agent that can be encapsulated in CC-GMS scaffolds to exert angiogenic effects in engineered tissue.

## Background

Skin loss remains a major healthcare problem around the world, especially in developing countries [1]. As such dermal defects do not heal spontaneously, scar formation due to full-thickness skin loss is inevitable unless skin substitutes are used [2]. The porous collagen/chitosan (CC) scaffold-based skin tissue engineering approach has become an important method for skin tissue repair and regeneration. Due to its natural origin, this scaffold has high biocompatibility and biodegradability. Implanted CC scaffolds could be used as a substrate for initial cell attachment and physical support for tissue guidance to accelerate wound healing in vivo [3,4]. However, a CC scaffold itself lacks normal vascular networks, and angiogenesis is slow after implantation in vivo. This scaffold may not be sufficient to induce rapid wound angiogenesis and regeneration at the initial stage of wound healing [5]. To accelerate angiogenesis, a CC scaffold could be combined with different types of microspheres as carriers for the controlled release of growth factors or other angiogenic factors. An extraordinary number of natural (i.e., collagen, alginate, and gelatin) and synthetic (i.e., poly(glycolic acid) and poly(L-lactic acid)) materials have been used as biomaterials in controlled-release applications [6]. Among these materials, gelatin, a natural polymer derived from collagen, is widely used. To load different bioactive factors, variations in the electrical and physical properties of gelatin microsphere (GMS)-based controlled-release systems can be achieved, depending on the fabrication method [7]. The combination of a GMS controlled drug delivery system with a skin tissue scaffold could be highly beneficial for wound angiogenesis and regeneration.

Angiogenic growth factors are often used in tissue engineering. However, growth factors have short biological half-lives. Platelet-derived growth factor (PDGF), isolated from platelets, cannot be detected in the circulation and has a half-life of less than 4 h when injected intravenously [8]. Moreover, the method of scaffold production and the surrounding in vivo environment may accelerate growth factor deactivation. Thus, there are limitations to maintaining therapeutic levels of growth factors at wound sites for healing periods of up to 2 weeks in the early period of angiogenesis.

*Panax notoginseng* (PNS), a well-known traditional Chinese medicine that has been extensively used for thousands of years, has been widely used in vitro and in vivo to encourage angiogenesis. Ginsenosides are the major active components of PNS. Ginsenoside Rg1, abundant in PNS, has a rigid steroidal skeleton with four transfused rings and two sugar moieties [9]. This ginsenoside is one of the most active ingredients in PNS and has a broad range of activities. Rg1 has estrogen-like activity and may represent a novel class of potent phytoestrogens [10]. Estrogen is known to directly modulate angiogenesis via effects on endothelial cells [11]. Rg1 has been demonstrated to have beneficial effects on ischemia-induced angiogenesis [12] and to enhance endothelial progenitor cell angiogenic potency [13]. These findings indicate the potential usefulness of ginsenoside Rg1 in angiogenesis and regeneration in skin tissue engineering.

Controlled release of Rg1 from a porous CC-GMS scaffold may be of effect in enhancing wound angiogenesis and healing. To date, there are few studies on Rg1 used in scaffolds, and the stability and activity of Rg1 remain unknown after its release from a CC-GMS scaffold. In this study, a porous CC-GMS scaffold for the controlled release of Rg1 was designed for angiogenesis and regeneration in skin tissue engineering. The Rg1 release kinetics were investigated. The activity of the released Rg1 was characterized by measuring human umbilical vein endothelial cells (HUVECs) proliferation, migration, tube formation, cell-cycle progression, apoptosis, and vascular endothelial growth factor (VEGF) expression. The optimum Rg1 concentration was also detected. Additionally, HUVECs were cultured on CC-GMS scaffolds to detect the scaffolds’ biocompatibility. The purpose of this study was to develop a new CC-GMS scaffold that can slowly release Rg1 and to evaluate the activity and stability of the released Rg1.

## Materials and methods

### Materials

Rg1 (purity > 98%) was purchased from the National Institute for the Control of Pharmaceutical and Biological Products (NICPBP, Beijing, China). A stock solution of Rg1 was prepared in sterile dimethyl sulfoxide (DMSO) (Sigma, Deisenhofen, Germany). VEGF and HUVECs were purchased from Life Technologies Corporation (Carlsbad, NM, USA). Medium 200 and Low Serum Growth Supplement (LSGS) were purchased from Invitrogen (Carlsbad, CA, USA). Fetal bovine serum was obtained from Gibco (Grand Island, NY, USA). An XTT Cell Proliferation Assay Kit (2, 3-bis-(2-methoxy-4-nitro-5-sulfophenyl)-2H-tetrazolium-5-carboxanilide), BD Matrigel™, a cell cycle kit, and a cell apoptosis kit were purchased from BD Biosciences (San Diego, CA, US). Transwell-24 well permeable supports (8.0 mm) were obtained from Corning Life Sciences. An anti-VEGF antibody was purchased from Abcam (Cambridge, MA, USA). Goat anti-rabbit IgG-FITC was purchased from Pierce (Rockford, IL, USA). The reagent 4′, 6-diamidino-2-phenylindole dihydrochloride (DAPI) was purchased from Sigma (St. Louis, MO, USA).

### Cell culture and seeding

HUVECs were expanded in Medium 200 supplemented with 10 ml of LSGS and used for experiments between passages 3 and 6 to ensure the genetic stability of the culture, according to the manufacturer’s instructions. The HUVECs were expanded in flasks in Medium 200 and 1% penicillin/streptomycin in a humidified incubator at 37°C with 5% CO_2_. Every 3–4 days, the HUVECs were passaged with 0.05% trypsin, and the medium was changed every other day.

### Preparation of CC scaffolds activated with GMSs containing Rg1/VEGF

Type A gelatin was used to fabricate GMSs following the method of emulsion-condensation cross-linking [14]. In brief, 5 ml of a gelatin solution (10%) and 100 ml of soybean oil (preheated to 45°C) were mixed together and stirred at 13,000 rpm for 10 min in a 45°C water bath. Ice water was then added while stirring to cool the gelatin solution and form microspheres. Next, the microspheres were washed with pre-cooled acetone to remove residual soybean oil. The precipitated microspheres were then cross-linked with a 1% glutaraldehyde solution at 37°C for 2 h, followed by the addition of glycine (Sigma) at room temperature for 30 min. The synthetic GMSs were washed with distilled water several times and dried at 37°C. The sizes and shapes of the microspheres were examined under a scanning electron microscope (SEM) (Philips XL30, Eindhoven, Netherlands). Microspheres with a diameter ranging from 10–50 μm were selected by sifting through mesh. The water content of the microspheres was calculated based on their volume variation after swelling in PBS at room temperature. The original Rg1 and VEGF solutions were diluted with distilled deionized water to a defined concentration. Incorporation of Rg1 or VEGF into the GMSs was achieved by adding one of the above solutions to 2 mg of freeze-dried GMSs and incubating for 2 h at room temperature to allow the solution to be impregnated into the dried microspheres. The Rg1 or VEGF solution was completely absorbed into the microspheres because the solution volume was much less than that theoretically required for the equilibrated swelling of microspheres. As a control, 20 μl of PBS was dropped onto 2 mg of freeze-dried GMSs to prepare Rg1-free, empty GMSs.

CC-GMS scaffolds were prepared using the freezing and lyophilizing method, with moderate modifications [15]. Briefly, type I collagen and chitosan were each dissolved in 0.5 M acetic acid to form 0.5% (w/v) solutions that were mixed at a ratio of 9:1 (v/v). GMSs containing different concentrations of Rg1, PBS, or VEGF were then homogeneously blended with the mixed solution. The cross-linking agent, a 0.1% (w/v) glutaraldehyde solution, was added, and the mixture was injected into a 12-well plate. The plates containing the scaffolds were frozen at −80°C overnight and then lyophilized for 24 h to form porous CC-GMS scaffolds. The scaffolds were washed several times with PBS and distilled water and were further treated with 75% ethanol before use.

The cumulative Rg1 released from the CC-GMS scaffolds in vitro was analyzed using a UV spectrophotometer at a wavelength of 208 nm, until release stopped. Finally, the released Rg1 was diluted to specific concentrations and then added to culture medium for further HUVEC culture experiments.

### In vitro study

The chemotactic effects of Rg1 on HUVECs were tested both for Rg1 released from the CC-GMS scaffold and for a standard Rg1 solution.

### Cell viability assessment by XTT assay

The viability of the cells was assessed by XTT assay. Briefly, HUVECs were seeded at a density of 1 × 10^4^ cells/well in a 96-well plate and cultured for 24 h. After cell attachment, low-serum culture medium (0.5% FBS) with different doses of released Rg1 (0–100 μg/ml) or 50 μg/ml standard Rg1 were added and cultured for 24, 48, or 72 h. Cells treated with 20 ng/ml VEGF served as positive control groups. Next, XTT solution was added to the wells (50 μl/well). After 4 h of incubation, the absorbance was measured with a microplate reader (Bio-Rad, Hercules, CA, USA) at 490 nm. Three duplicate wells from at least three independent experiments were tested.

### Transwell migration

The effect of Rg1 on HUVEC invasion was measured using a 6.5 mm Transwell tissue culture insert with an 8.0 μm polycarbonate membrane and a 24-well companion plate. The upper side of the membrane was pre-coated with 1:50 (v/v) Matrigel. In total, 2 × 10^4^ HUVECs were resuspended in low-serum medium (200 μl) and seeded onto the culture inserts in triplicate. The inserts were then deposited into the 24-well companion plate along with 600 μl of low-serum medium containing different concentrations of released Rg1 (0–100 μg/ml), standard Rg1, or VEGF. Next, 24 h after HUVEC seeding, the inserts were removed, fixed in 20% ethanol, and then washed with PBS. Noninvasive cells on the upper surface of the membrane were removed. Cells that migrated to the bottom well through the porous membrane were stained with a 0.1% crystal violet solution for 20 min, photographed at 100 × magnification, and counted using Image-Pro Plus software (v.6.0; Media Cybernetics, Silver Spring, MD). The percent increase in migration in each experimental group was compared with migration in the control group.

### Tube formation assay and quantification

In total, 1 × 10^5^ HUVECs were cultured in 48-well plates with a layer of 10 mg/ml Matrigel diluted with 1:1 (v/v) serum-free medium. The plates pre-coated with Matrigel were then incubated at 37°C for 60 min. HUVEC suspensions were cultured in culture medium with different concentrations of released Rg1, standard Rg1, or VEGF. The plates were incubated for 8 h at 37°C. Tubular structures were dyed with calcein-AM, photographed (100 ×), and examined using Image J software (US National Institutes of Health, http://rsb.info.nih.gov/ij/). The percent of tube formation in each experimental group was compared with tube formation in the control group.

### Apoptosis determination by annexin V-FITC assay

In brief, 5 × 10^5^ cells/well were seeded onto six-well culture plates. After 24 h of cell attachment and 24 h of serum starvation, the cells were exposed to fresh medium with different concentrations of released Rg1, standard Rg1, or VEGF. After 24 h of culture, the cells were rinsed with PBS, trypsinized, and centrifuged for 5 min at 2,000 rpm. The cells were then resuspended in 500 μl of Annexin V Binding Buffer, which was provided in the Annexin V-FITC Kit (KeyGEN, Beijing, China). Next, 5 μl of annexin V-FITC and 5 μl of propidium iodide (PI) were added to the test tube. The cells were incubated at room temperature for 15 min and protected from light. The samples were then analyzed with a BD FACScan analyzer.

### Cell-cycle progression

Cells were seeded onto six-well culture plates at a density of 5 × 10^5^ cells/well. After 24 h of cell attachment and 24 h of serum starvation, different concentrations of released Rg1, standard Rg1, or VEGF were added to the culture medium for 24 h. Both floating and attached cells were collected and fixed in 500 μl of ice-cold 75% ethanol overnight and washed three times in PBS. The cells were then treated with 100 μl of RNase A at 37°C for 30 min, stained with 400 μl of PI at 4°C for 30 min, and stored in the dark. The fluorescence of PI in HUVECs was measured using a BD FACScan analyzer. The wavelength of laser excitation was set at 488 nm, with an emission wavelength at 590 nm. In total, 20,000 cells were analyzed for each group. The percentages of cells in the G0/G1 phase, S phase, and G2/M phase were counted using GraphPad Prism 5.0 software (GraphPad Prism Software, San Diego, CA, US).

### Immunocytofluorescence staining of VEGF

Immunocytofluorescence (ICF) staining was performed to demonstrate VEGF expression in HUVECs. HUVECs were first cultured in 24-well plates. After 24 h of cell attachment, different concentrations of released Rg1, standard Rg1, or VEGF were added to the culture medium for 24 h. The HUVECs were washed with PBS three times and fixed with 4% chilled paraformaldehyde for 20 min. The HUVECs were washed three times in PBS, followed by a blocking step using 5% bovine serum albumin (BSA) for 20 min. A mouse monoclonal anti-VEGF antibody was then added and incubated overnight, followed by incubation with FITC-conjugated goat anti-mouse IgG secondary antibody for 1 h at room temperature in the dark. Additionally, the cell nuclei were stained with DAPI for 30 min at room temperature in the dark. After all of the steps, the cells were observed and photographed using a fluorescence microscope (Leica, Germany). Images were acquired and processed using Image-Pro Plus software [16].

### Cell culture on CC-GMS scaffolds

Composite scaffolds for in vitro cell growth were sterilized by exposure to UV light for 30 min on each side. Aliquots of 20 μl of HUVECs were seeded onto CC-GMS scaffolds containing different concentrations of Rg1 or VEGF at a density of 5 × 10^4^ cells per scaffold. The scaffolds were left undisturbed in an incubator for 2 h at 37°C to allow cell attachment, after which an additional 1 ml of low-FBS medium was added to each well. The cells were cultured for 7 days, and the medium was changed every 3 days. Afterward, the scaffolds were first washed with PBS three times and then were fixed in 4% paraformaldehyde overnight. After washing with PBS three times, the scaffolds were stained with 100 μg/ml DAPI for 30 min at 37°C, followed by FITC staining for 20 min. The scaffolds were then photographed using a laser scanning confocal microscope (Leica, Germany).

### Statistical analysis

The data were expressed as the mean ± standard deviation (SD) and were analyzed by one-way ANOVA and Tukey’s tests to determine the level of significance. A p value < 0.05 was considered to be significant, and p < 0.01 was considered to be highly significant.

## Results

### Fabrication of GMSs and CC-GMS scaffolds

**Figure 1:**
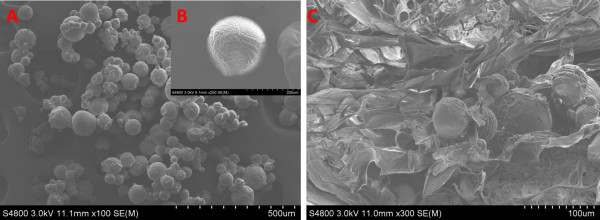
**Scanning electron micrographs of GMSs and the CC-GMS scaffold. A-B**: Scanning electron micrographs of GMSs. **C**: Scanning electron micrographs of the CC-GMS scaffold. The average inner pore size of the CC-GMS scaffold was 110 ± 12 μm, and the diameters of the GMSs were between 10 and 50 μm.

Figure 1 demonstrates the morphology of the GMSs and CC-GMS scaffolds. The GMSs were round, with diameters between 10 and 50 μm (Additional file 1). The CC-GMS scaffold demonstrated a three-dimensional porous structure with a pore size of 110 ± 12 μm and a porosity of 92% ± 0.4%. The diameter of the CC-GMS scaffolds was approximately 2 cm, with a thickness of 2 ± 0.2 mm. The CC-GMS scaffold possessed interconnected structures after the GMSs were distributed evenly. The degradation rate of GMS and CC-GMS scaffold were 21.67 ± 3.05 days and 23.33 ± 1.52 days respectively.

### Release of Rg1 from CC-GMS scaffold

**Figure 2:**
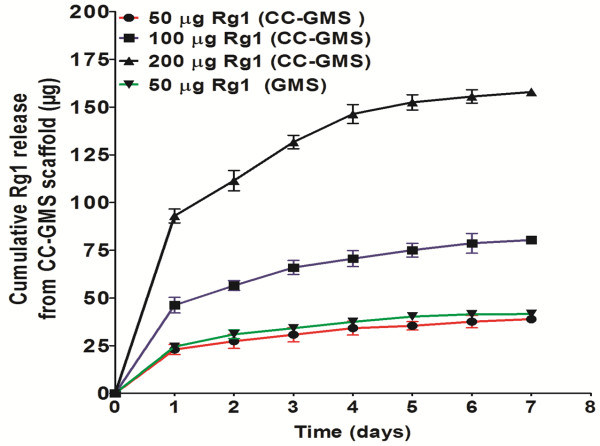
**Release of Rg1 from the CC-GMS scaffold and GMS.** The effects of loading content on Rg1 release: (●) 50 μg, (■) 100 μg, and (▲) 200 μg of Rg1 in CC-GMS scaffolds and (▼) represent 50 μg Rg1 in GMS.

The cumulative Rg1 released from the CC-GMS scaffold and the GMSs is shown in Figure 2. Initial burst release was observed both from the CC-GMS scaffold and the GMSs during the first day, followed by slow release for up to 7 days, followed by a leveling off. The initial release of Rg1 (50, 100, or 200 μg) from the CC-GMS scaffold was 46%, 46.26%, and 46.5%, compared respectively with each total Rg1 dosage. The initial release of Rg1 (50 μg) from the GMSs was 49.667%. Then the amount released from both systems increased stably and slowly. After 7 days, the total release was 77.86%, 80.46%, and 78.96%, respectively from the CC-GMS scaffold and 83.27% from the GMSs. As the Rg1 loading content increased, the release rate increased correspondingly.

### Effects of Rg1 on HUVEC proliferation

The loading of Rg1 into the CC-GMS scaffold may alter the activity of Rg1. In this study, chemotactic activity was measured to examine the stability and activity of the Rg1 that was released from the CC-GMS scaffold.

**Figure 3:**
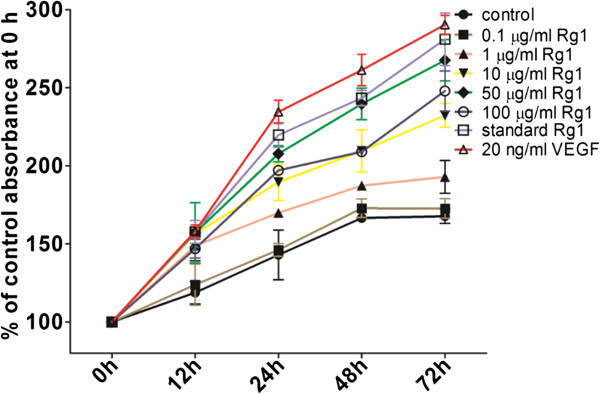
**Effects of the released Rg1 on HUVEC proliferation.** Cell proliferation was assessed using the XTT assay after 12, 24, 48 or 72 h of treatment with different concentrations of released Rg1, 50 μg/ml standard Rg1, or 20 ng/ml VEGF. The results are expressed as the percent of cell proliferation compared with the control at 0 h. The data are expressed as the mean ± SD from three individual experiments. *p < 0.05 compared with the control group.

The effect of the released Rg1 on the proliferation of HUVECs was evaluated by XTT assay. As shown in Figure 3, Rg1 increased the proliferation of HUVECs in a dose- and time-dependent manner, with statistical significant proliferation observed at 50 μg/ml and at a time point of 72 h, with proliferation in 267.59% of HUVECs. The proliferative effect of 50 μg/ml released Rg1 was slightly lower than the effect of VEGF (290.401%) or 50 μg/ml standard Rg1 (281.085%) but higher than the effect of 100 μg/ml released Rg1 (247.955%).

### Effect of Rg1 on HUVEC migration

**Figure 4:**
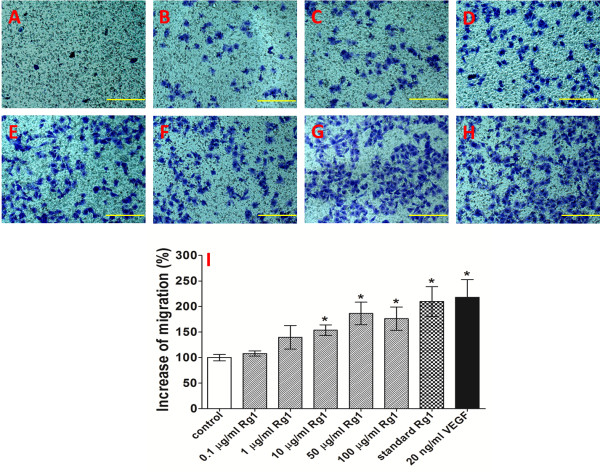
**Effect of the released Rg1 on HUVEC migration.** Observation of the effect of the released Rg1 on HUVEC migration after 24 h using a Transwell culture insert. **A**: Control group (untreated); **B**: 0.1 μg/ml released Rg1; **C**: 1 μg/ml released Rg1; **D**: 10 μg/ml released Rg1; **E**: 50 μg/ml released Rg1; **F**: 100 μg/ml released Rg1; **G**: 50 μg/ml standard Rg1; and **H**: 20 ng/ml VEGF. **I**: The percentage increase in HUVEC migration was compared with migration in the control group (untreated). Photographed by microscope at 200 × .The data are expressed as the mean ± SD. Significant differences compared with controls are presented (*p < 0.05).

The influence of the released Rg1 on HUVEC migration was determined by measuring the number of migrated cells, as shown in Figure 4. Compared with the control group, the released Rg1 groups showed a dose-dependent increase in the migration of HUVECs. Rg1 reached a maximum effect at a concentration of 50 μg/ml (Figure 4I). When the concentration reached 100 μg/ml, the migrated cell number did not increase. VEGF at 20 ng/ml had a 14.6% greater pro-migration effect than did 50 μg/ml Rg1. Meanwhile, the released Rg1 at 50 μg/ml showed little decrease (11.1%) compared with 50 μg/ml standard Rg1. There was no statistical difference between the 50 μg/ml standard Rg1 group and the VEGF group regarding HUVEC migration.

### Effect of Rg1 on Matrigel-induced tube formation

**Figure 5:**
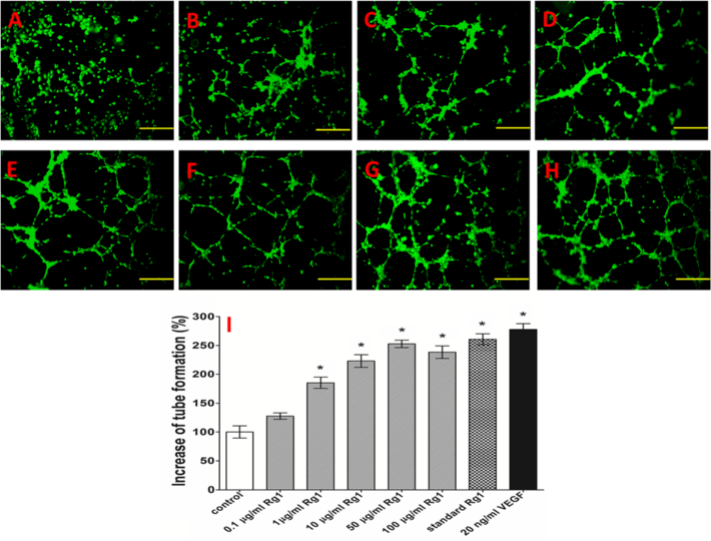
**Effect of Rg1 on HUVEC tube formation.** In total, 1 × 10^5^ HUVECs were seeded on Matrigel-coated 48-well culture plates and treated with released Rg1, standard Rg1, or VEGF. After 8 h, tubular structures were photographed (100 ×). **A**: Control group (untreated); **B**: 0.1 μg/ml released Rg1; **C**: 1 μg/ml released Rg1; **D**: 10 μg/ml released Rg1; **E**: 50 μg/ml released Rg1; **F**: 100 μg/ml released Rg1; **G**: 50 μg/ml standard Rg1; and **H**: 20 ng/ml VEGF. **I**: The percentage increase in tube formation was compared with tube formation in the control group (untreated). Photographed by microscope at 100 ×. The data are expressed as the mean ± SD. Significant differences compared with controls are presented (*p < 0.05).

To test the effect of the released Rg1 on HUVEC tube formation, a Matrigel model was used in this study. HUVECs did not form tubes, at least at a seeding density of 50,000 cells per well. As shown in Figure 5, the number of branching points was counted and then compared with that of the control group. The released Rg1, the standard Rg1, and VEGF all triggered significant increases in the number of elongated and robust tube-like structures. The released Rg1 induced cell tube formation in a dose-dependent manner, and the most effective concentration was 50 μg/ml. When the Rg1 concentration was above 1 μg/ml, the stimulation was found to be statistically significant compared with that of the blank control (p < 0.05). The standard Rg1 group exhibited 6.18% lower activity than did the VEGF group. However, the remaining activity of 50 μg/ml released Rg1 remained approximately the same as that of standard Rg1.

### Cell cycle and apoptosis analysis

**Figure 6:**
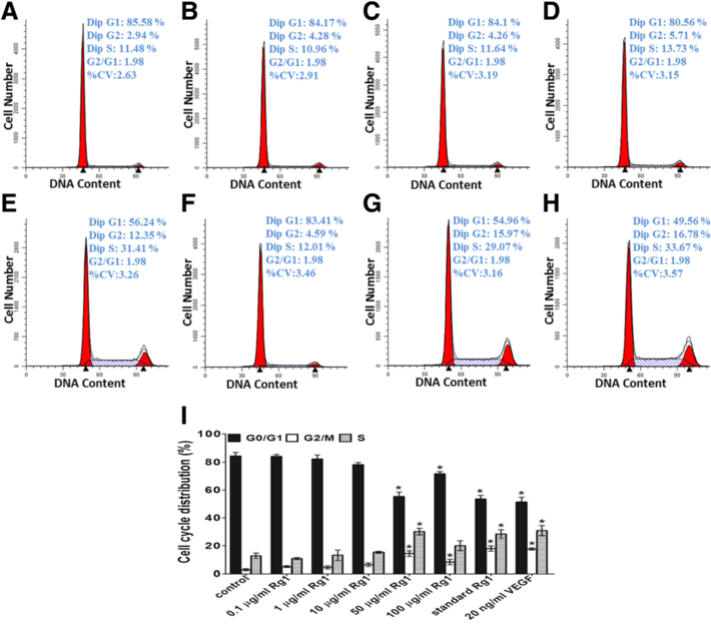
**Cell cycle of HUVECs after 24 h of treatment, as detected by flow cytometry. A**: Control group (untreated); **B**: 0.1 μg/ml released Rg1; **C**: 1 μg/ml released Rg1; **D**: 10 μg/ml released Rg1; **E**: 50 μg/ml released Rg1; **F**: 100 μg/ml released Rg1; **G**: 50 μg/ml standard Rg1; and **H**: 20 ng/ml VEGF. The percentage of cells in the G0/G1, S, and G2/M phases are indicated on the upper right side. **I**: The percentage of cells residing in the G0/G1, S, and G2/M phases. The data are expressed as the mean ± SD. Significant differences compared with controls are presented (*p < 0.05).

To determine the effect of the released Rg1 on the HUVECs’ cell cycle, a cell cycle analysis was performed. Incubation with either Rg1 or VEGF resulted in increased cell-cycle progression (Figure 6). Rg1 significantly increased the number of HUVECs in the proliferative phase (S and G2/M phases) and decreased the number in the resting phase (G0/G1 phase) in a dose-dependent manner. The control group displayed a low percentage of proliferation (S phase, 12.78%, and G2/M phase, 2.84%), whereas the percentage of cells in the S phase increased to 30.23% and in the G2/M phase increased to 14.46% with 50 μg/ml Rg1 treatment. Compared with 1, 10 and 100 μg/ml Rg1 group, 50 μg/ml Rg1 group had statistical significant differences both in S phase and G2/M phase. In the VEGF group, 30.94% of cells were in the S phase, and 17.69% were in the G2/M phase. There was no statistical difference between 50 μg/ml Rg1 group and VEGF group. The standard Rg1 group showed a similar effect as the 50 μg/ml released Rg1 group, with 28.45% cells in the S phase and 18.02% in the G2/M phase. These data suggest that Rg1 induces cell proliferation by increasing the proportion of cells in the S and G2/M phases and that the released Rg1 did not lose its activity.

**Figure 7:**
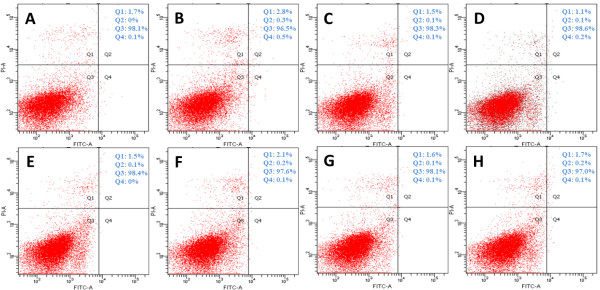
**Cell apoptosis of HUVECs after 24 h of treatment, as detected by flow cytometry.** Representative images of cell apoptosis. **A**: Control group (untreated); **B**: 0.1 μg/ml released Rg1; **C**: 1 μg/ml released Rg1; **D**: 10 μg/ml released Rg1; **E**: 50 μg/ml released Rg1; **F**: 100 μg/ml released Rg1; **G**: 50 μg/ml standard Rg1; and **H**: 20 ng/ml VEGF. The percentage of cells in each percentage are indicated on the upper right side.

Annexin V-FITC/PI staining was used to determine the extent of HUVEC apoptosis. As shown in Figure 7, the released Rg1 groups, the VEGF group, and the standard Rg1 group did not show an increase in the apoptosis of HUVECs. There were few annexin V-FITC- and PI-positive cells. These results demonstrate that Rg1 has little toxicity toward HUVECs at the tested concentrations and that CC-GMS scaffold degradation did not increase the toxicity of the released Rg1.

### Effect on VEGF expression

**Figure 8:**
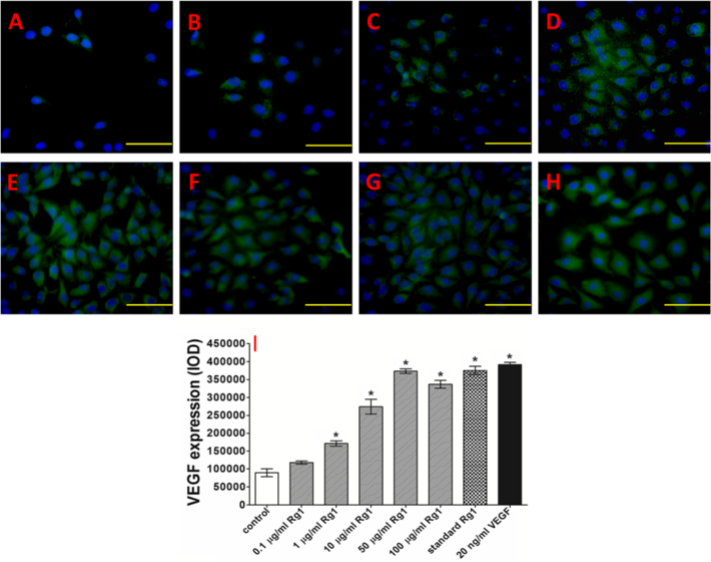
**VEGF expression of in vitro-cultured HUVECs.** ICF analysis of the effect of the released Rg1 on the VEGF expression of HUVECs after 24 h of treatment. **A**: Control group (untreated); **B**: 0.1 μg/ml released Rg1; **C**: 1 μg/ml released Rg1; **D**: 10 μg/ml released Rg1; **E**: 50 μg/ml released Rg1; **F**: 100 μg/ml released Rg1; **G**: 50 μg/ml standard Rg1; and **H**: 20 ng/ml VEGF. **I**: The relative counts of VEGF-positive cells, as determined by ICF staining for VEGF. IOD: Integral optical density. Significant differences compared with controls are presented (*p < 0.05). Photographed by microscope at 200 × .

The expression of VEGF, which normally creates new blood vessels, was further assessed by an ICF assay after 24 h of incubation. As shown in Figure 8, both Rg1 and VEGF caused an increase in VEGF expression, and the VEGF expression of HUVECs increased along with the increase in the released Rg1 concentration. In the 50 μg/ml Rg1 group, the VEGF expression was most significant, at approximately 4.18 times of the expression in the control group. The VEGF expression of the VEGF group and the standard Rg1 group was 4.38 and 4.20 times of the control group’s expression, respectively. Compared with standard Rg1, the released Rg1 at 50 μg/ml showed a similar effect on VEGF expression. These findings demonstrated that Rg1 can induce VEGF expression, which is of great importance in the early stage of angiogenesis.

### HUVEC proliferation on CC-GMS scaffold

**Figure 9:**
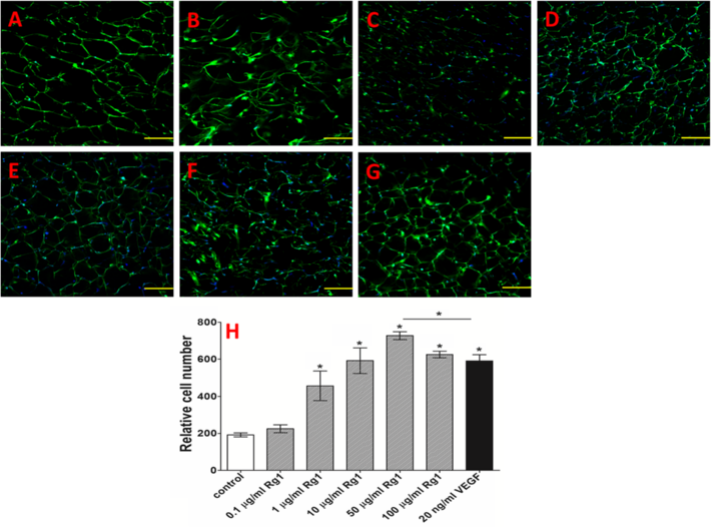
**Observation of cell attachment to and proliferation in CC-GMS scaffolds by LSCM.** After 7 days of cell culture in CC-GMS scaffolds, the scaffolds and cell nuclei were stained with FITC and DAPI, respectively, and observed by LSCM. **A**: Control group (untreated); **B**: 0.1 μg/ml released Rg1; **C**: 1 μg/ml released Rg1; **D**: 10 μg/ml released Rg1; **E**: 50 μg/ml released Rg1; **F**: 100 μg/ml released Rg1; and **G**: 20 ng/ml VEGF. **H**: The relative counts of cells attached to the CC-GMS scaffold. Green and blue represent the CC-GMS scaffold and cell nuclei, respectively, photographed by a microscope at 200 ×. Significant differences compared with controls are presented (*p < 0.05).

Figure 9 shows laser scanning confocal microscopy (LSCM) images of proliferated HUVECs on the Rg1-loaded CC-GMS scaffold after 7 days of cultivation. The scaffold was brittle in its dried state but provided good malleability after being wetted with culture medium. The HUVECs were well attached and proliferated in a multilayered phase over the CC-GMS scaffold. Compared with proliferation in the vehicle group, enhanced HUVEC proliferation was observed within both the Rg1- and the VEGF-loaded CC-GMS scaffolds. Moreover, after 7 days of cultivation, the 50 μg/ml Rg1 group showed 18.84% more HUVEC proliferation than did the VEGF group. These results demonstrated that the porous CC-GMS scaffold could be utilized as a skin tissue substitute with the ability to regulate Rg1 release and the potential to increase cellular growth.

## Discussion

VEGF is an endothelial cell-specific mitogen and an angiogenesis inducer. VEGF promotes the growth of vascular endothelial cells from the arteries, veins, and lymphatics [17]. VEGF is also a survival factor for endothelial cells, as it prevents the endothelial apoptosis induced by serum starvation. This prevention is achieved by mediating the phosphatidylinositol 3-kinase (PI3K)/Akt pathway [18] and inducing expression of the anti-apoptotic proteins Bcl-2, A1, XIAP, and survivin in endothelial cells [19,20]. As a functional ligand of the glucocorticoid receptor (GR), Rg1 has been shown to increase the phosphorylation of GR and the activities of PI3K, Akt/protein kinase B, and eNOS in HUVECs [21]. PI3K/Akt signaling has been found to be significantly important in HUVEC growth, survival, protein synthesis, and angiogenesis [22]. Consistent with this pathway, similar to VEGF, standard Rg1 at 50 μg/ml stimulated significant HUVEC proliferation, migration, and tube formation, with increases of 267.59% (72 h), 186.42%, and 252.73%, respectively, compared with the vehicle control group. This finding is in accordance with reports that Rg1 promotes HUVEC proliferation, migration, and tube formation [23].

Moreover, as expected, both Rg1 and VEGF promoted HUVEC transition from G0/G1 to G2/M. Treatment of HUVECs with standard Rg1 or VEGF for 24 h induced a significant percentage of cells to enter the S and G2/M phases (28.45% and 30.94%, respectively, in the S phase and 18.02% and 17.69%, respectively, in the G2/M phase) compared with the control (12.78% in the S phase and 2.84% in the G2/M phase; Figure 6). Additionally, after 24 h of treatment, Rg1 did not increase the proportion of apoptotic cells (Figure 7), and all of the groups were similar. In support of our data, it has been reported that the activation of PI3K and PKB leads to FOXO3a phosphorylation and sequestration in the cytoplasm, thereby reducing growth arrest and DNA damage-inducible 45a expression, thus activating G2/M progression [24].

Because VEGF is known to be a key activator of angiogenesis, we examined whether Rg1 could upregulate VEGF production. We found that VEGF production was significantly elevated in response to Rg1 stimulation, as determined by an ICF assay (Figure 8). In support of our data, Rg1 has been reported to be a potent stimulator of VEGF expression in HUVECs, and importantly, this induction is mediated through a PI3K/Akt- and β-catenin/T-cell factor-dependent pathway via the GR [25]. These results suggest that Rg1 promotes HUVEC survival, proliferation, migration, tube formation, cell-cycle progression, and VEGF expression and that these effects are dependent on the PI3K/Akt pathway.

In this study, GMSs were used as carriers. The GMS release rate could be controlled by various cross-linking conditions, including the cross-linking agent type, density, and reaction period [26]. The cross-linking conditions were 1% glutaraldehyde solution at 37°C for 2 h. Under these conditions, the initial burst release of Rg1 was approximately 46% (Figure 2). Rapid release at the initial step and maintenance of a proper concentration at the local site are favorable for bioreagent delivery [27]. The sustained release test in this study (Figure 2) clearly demonstrated that Rg1 was gradually released from the Rg1-impregnated CC-GMS scaffold. Moreover, the release rate of Rg1 from the CC-GMS scaffold varied with the loading content. The release rate increased proportionally as the loading content increased. An initial Rg1 burst release may properly induce HUVEC attachment and proliferation, and the following steady release of Rg1 efficiently stimulates HUVEC migration and tube formation, thus leading to vascular structure formation. Incorporation of GMSs into the CC-GMS scaffold was thus required to attain steady Rg1 release.

Ginsenoside Rg1 consists of a gonane steroid nucleus and has 17 carbon atoms arranged in four rings [28]. The specific chemical structure of Rg1 consists of a high degree of ring structure, which makes its flexibility very limited. The ring structure is known to be inherently stable [29]. The Rg1 released from the CC-GMS scaffold showed nearly the same tube formation activity (96.06%) as did the standard Rg1 solution. This result revealed that the Rg1 in the CC-GMS scaffold maintained its biological activity and that the fabrication procedure did not affect the stability of Rg1. In addition, the effect of the Rg1 released from the CC-GMS scaffold showed that Rg1 exerts its effect in a dose-dependent manner. Additionally, the concentration of Rg1 (50 μg/ml) used in this study is the optimal stimulatory dose for HUVECs, with responses slightly decreasing at higher doses. This phenomenon may be due to the effect of the cytotoxicity of ginsenoside Rg1 when the concentration is increased to 100 μg/ml. Additionally, the above results revealed that the release rate of Rg1 can be controlled by varying the initial loading content of Rg1 to attain optimal therapeutic efficacy for wound tissue regeneration.

The Rg1-loaded CC-GMS scaffold induced significantly high cell attachment and proliferation, which indicated good cellular adaptability. Steady stimulation by the Rg1 from the CC-GMS scaffold might cause rapid cell proliferation within the scaffold. At 7 days after HUVEC culture on the CC-GMS scaffold, the Rg1-mediated effect on HUVEC attachment was approximately 18.84% higher than the effect of VEGF (Figure 9). These results indicated that the chemical stability of Rg1 was significantly better than that of VEGF. It is known that proteins are often unstable outside their native environments. The occurrence of protein denaturation may be attributed to a variety of factors, such as pH, buffer species, and temperature. The half-life of VEGF was less than 10 h. In contrast, the activities of Rg1 remained approximately the same. These results demonstrated that the CC-GMS scaffold can be utilized as a skin tissue substitute material with the ability to regulate Rg1 release and the potential to stimulate cellular growth. It is anticipated that different types of cells will freely migrate into Rg1-loaded CC-GMS scaffolds when applied to a skin defect to improve skin tissue angiogenesis and regeneration efficacy.

## Conclusion

Rg1-loaded CC-GMS scaffolds may control Rg1 release, serve as a physical scaffold for cell proliferation, and promote angiogenesis. The Rg1 in the scaffold retained its biological activity, and the Rg1 released from the CC-GMS scaffold enhanced HUVEC proliferation, migration, tube formation, cell-cycle progression, and VEGF expression. The Rg1-loaded CC-GMS scaffold might be a valuable modality in skin tissue engineering-based angiogenesis and regenerative therapy.

## Abbreviations

PNS: *Panax notoginseng*; CC-GMS: Collagen/chitosan-gelatin microsphere; HUVECs: Human umbilical vein endothelial cells; VEGF: Vascular endothelial growth factor; CC: Collagen/chitosan; GMS: Gelatin microsphere; PDGF: Platelet-derived growth factor; NICPBP: National Institute for the Control of Pharmaceutical and Biological Products; DMSO: Dimethyl sulfoxide; LSGS: Low serum growth supplement; XTT: 2, 3-bis-(2-methoxy-4-nitro-5-sulfophenyl)-2H-tetrazolium-5-carboxanilide; DAPI: 4′, 6-diamidino-2-phenylindole dihydrochloride; SEM: Scanning electron microscope; ICF: Immunocytofluorescence; BSA: Bovine serum albumin; LSCM: Laser scanning confocal microscopy; PI3K: Phosphatidylinositol 3-kinase; GR: Glucocorticoid receptor.

## Competing interests

The authors declare that they have no competing interests.

## Authors’ contributions

YZ and ZF carried out the in vitro experiments and drafted the manuscript. CY carried out the scanning electron microscope examination. YJ participated in the immunocytofluorescence staining. XH and XW participated in the design of the study and performed the statistical analysis. CH conceived of the study, and participated in its design and coordination and helped to draft the manuscript. All authors read and approved the final manuscript.

## Supplementary Material

Additional file 1: Figure S1 Micrographs of GMSs at 100 ×. Click here for file
