# Supplementary figures and images for: In vitro evaluation of Panax notoginseng Rg1 released from collagen/chitosan-gelatin microsphere scaffolds for angiogenesis
Source: Biomed Eng Online. 2013 Dec 31;12:134. doi: 10.1186/1475-925X-12-134 (PMC3937171; doi:10.1186/1475-925X-12-134)

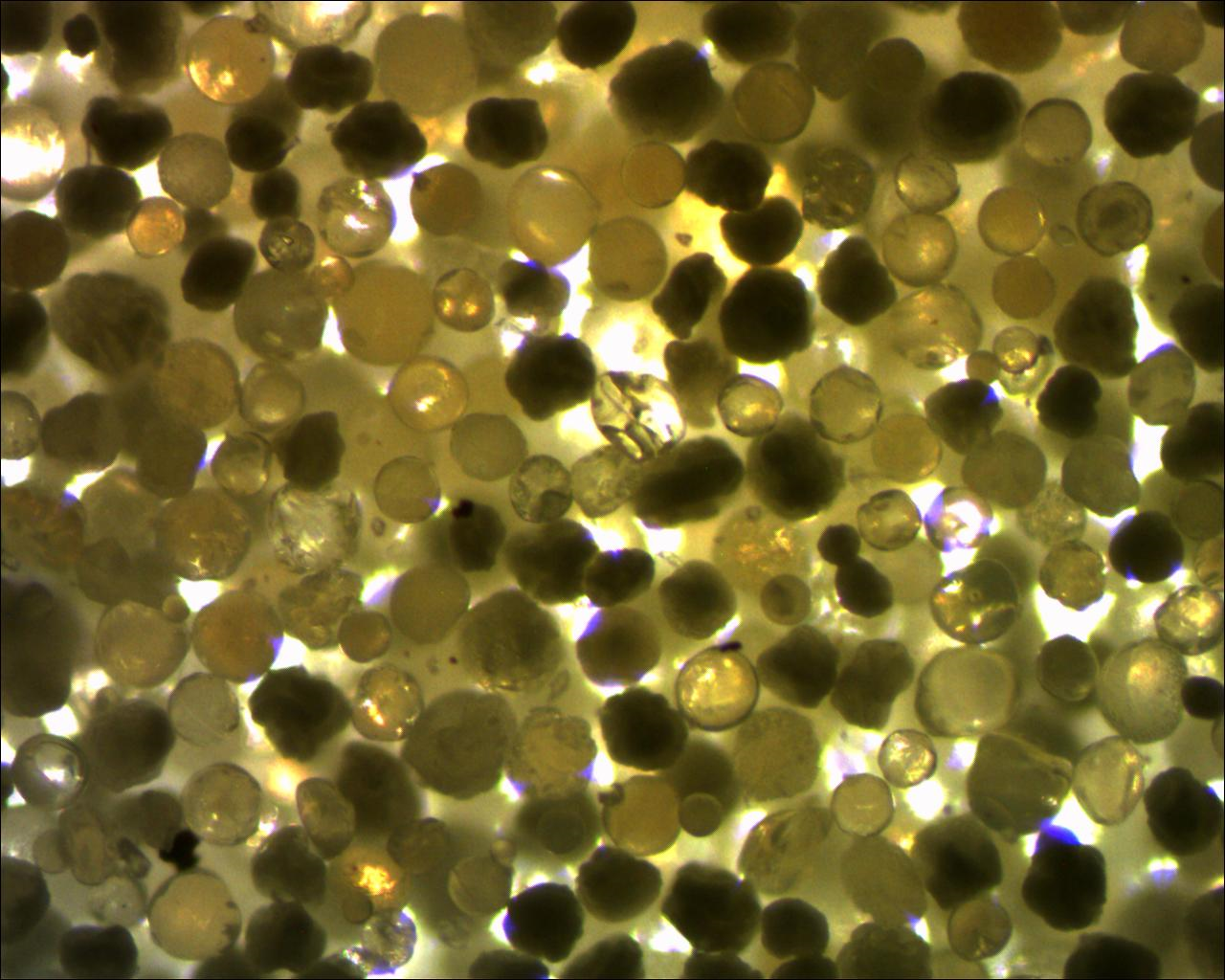

Supplement: Additional file 1: Figure S1 — Micrographs of GMSs at 100 ×. [file 1475-925X-12-134-S1.png]
